# Functional Basis of Microorganism Classification

**DOI:** 10.1371/journal.pcbi.1004472

**Published:** 2015-08-28

**Authors:** Chengsheng Zhu, Tom O. Delmont, Timothy M. Vogel, Yana Bromberg

**Affiliations:** 1 Department of Biochemistry and Microbiology, Rutgers University, New Brunswick, New Jersey, United States of America; 2 Environmental Microbial Genomics, Laboratoire Ampere, École Centrale de Lyon, Université de Lyon, Ecully, France; 3 Institute for Advanced Study, Technische Universität München, Garching, Germany; University College London, UNITED KINGDOM

## Abstract

Correctly identifying nearest “neighbors” of a given microorganism is important in industrial and clinical applications where close relationships imply similar treatment. Microbial classification based on similarity of physiological and genetic organism traits (polyphasic similarity) is experimentally difficult and, arguably, subjective. Evolutionary relatedness, inferred from phylogenetic markers, facilitates classification but does not guarantee functional identity between members of the same taxon or lack of similarity between different taxa. Using over thirteen hundred sequenced bacterial genomes, we built a novel function-based microorganism classification scheme, functional-repertoire similarity-based organism network (FuSiON; flattened to *fusion*). Our scheme is phenetic, based on a network of quantitatively defined organism relationships across the known prokaryotic space. It correlates significantly with the current taxonomy, but the observed discrepancies reveal both (1) the inconsistency of functional diversity levels among different taxa and (2) an (unsurprising) bias towards prioritizing, for classification purposes, relatively minor traits of particular interest to humans. Our dynamic network-based organism classification is independent of the arbitrary pairwise organism similarity cut-offs traditionally applied to establish taxonomic identity. Instead, it reveals natural, functionally defined organism groupings and is thus robust in handling organism diversity. Additionally, *fusion* can use organism meta-data to highlight the specific environmental factors that drive microbial diversification. Our approach provides a complementary view to cladistic assignments and holds important clues for further exploration of microbial lifestyles. *Fusion* is a more practical fit for biomedical, industrial, and ecological applications, as many of these rely on understanding the functional capabilities of the microbes in their environment and are less concerned with phylogenetic descent.

## Author Summary

Taxonomic classification of microorganisms according to similarity is important for industrial and clinical applications where close relationships imply similar uses and/or treatments. Current microbial taxonomy is phylogeny-guided, *i*.*e*., the organisms are grouped based on their evolutionary relationships, defined by vertical inheritance of genetic information from mother to daughter cells. Microbes, however, are capable of horizontal gene transfer (HGT). Thus, the current taxonomic assignments cannot guarantee genome-encoded molecular functional similarity; *i*.*e*. two microbes of the same taxonomic group inhabiting different environments may be very different—just as your cousin may be more different from you than your unrelated best friend. Our work establishes a computational framework for comparison of microorganisms based on their molecular functionality. In our functional-repertoire similarity-based organism network (FuSiON; flattened to *fusion*) representation, organisms can be consistently assigned to groups based on a quantitative measure of their functional similarities. Our approach highlights the specific environmental factor(s) that explain the functional differences between groups of microorganism. *Fusion* is a more practical choice for biomedical, industrial, and ecological applications, as many of these rely on understanding the functional capabilities of the microbes in their environment.

## Introduction

In biology, the field of taxonomy is tasked with describing, naming, and classifying organisms; the latter according to some metrics of similarity. Van Leeuwenhoek’s observation of microscopic organisms launched centuries of classification based on morphology and physiology [1]. Since the 1960’s, DNA-DNA hybridization (DDH) [2] has been the ‘gold standard’ for bacterial species demarcation. The current polyphasic species definition requires a DDH value >70%, as well as shared phenotypic characteristics, to assign two bacteria to the same species [3]. Recent emergence of high-throughput genomic sequencing [4] highlighted the importance of genomic similarity in bacterial taxonomy. For example, studies have shown that the average genome nucleotide identity (ANI) classifies bacterial species as well as DDH values [5]. These new metrics also revealed previously unseen organismal relationships, highlighting the dynamic state of the prokaryotic taxonomy. As there is no one *true* taxonomy, subjectivity is a factor in comparing and contrasting conflicting classifications. Furthermore, special human interest, *e*.*g*. pathogenicity, and the desire to conserve existing naming conventions add to the inconsistency.

Today, prokaryotic taxonomy relies heavily on phylogenetics. However, there are non-phylogenetic alternatives for classification. Phenetics [6], for example, classifies organisms based on similarity regardless of shared ancestry. The definition of the term “similarity” is fluid, but in its broadest sense implies a comparison of organism phenotypes, including their molecular functional capabilities. It is important to note that though both phylogeny-based taxonomy (cladistics) and phenetics can be used to investigate bacterial relationships, the questions that they try to answer are different. The task of phylogeny is reconstructing organismal evolutionary *history*–think *Tree of Life* [7,8] efforts. Phenetics, on the other hand, clusters organisms into *currently* consistent classes on the basis of observable traits. Closely related organisms are often phenotypically similar. However, the order of evolutionary descent does not directly translate to classification–just as whales are more related to cows than to fish, despite the obvious morphological, environmental, and functional similarities to the latter.

The current NCBI Taxonomy [9], a trusted computationally accessible resource, largely follows Bergey’s Manual of Systematic Bacteriology [10]. Bergey’s Manual is a framework of prokaryotic taxonomy built around a backbone of 16S rRNA-derived phylogeny, which is used to find “unifying concepts of bacterial taxa [leading] to greater taxonomic stability and predictability.” However, as physiology and morphology are also relevant to classification, the boundaries between different taxa are often subjective and controversial [10]. Additional techniques, *e*.*g*. multi-locus sequence analysis (MLSA) [11], are often used to compensate for the lack of 16S rRNA phylogeny resolution [12]. For the (even highly accurate) computational organism classification methods [13] this taxonomic flexibility contributes to inconsistent assignments.

Due to the absence of sexual reproduction and the presence of horizontal gene transfer (HGT), speciation is not strictly defined in prokaryotes. Therefore, the goal of greater classification *stability and predictability* could be better achieved via phenetically clustering organisms on the basis of quantifiable similarity of their molecular function capabilities. In early studies, Enterotubes, a one-stop shop for dozens of biochemical tests, were used to accurately classify *Enterobacteriaceae* [14]; however, these could not be applied to other organisms. Gram staining, on the other hand, could broadly typify bacteria, but lacked in taxonomic resolution. In general, biochemical/physiological tests only reflect a small portion of bacterial functionality–as many as three hundred tests would only access 5–20% of the bacterial functional potential [10]. Cheaper genome sequencing and advanced computational methods offer a different route for measuring bacterial functional capabilities.

Most of the molecular functionality of one bacterium, its functional repertoire, is carried by its proteome, the set of all proteins encoded by its genes. Note that while plasmid encoded proteins are also part of the proteome, for reasons discussed later in the manuscript, here we only focus on the proteins encoded on the bacterial chromosome. The current taxonomy usually reflects either the phenotypic manifestations of functional repertoire subsets (morphology, physiology) or high-level repertoire interpretations (*e*.*g*. DDH). Ideally, however, comparison between bacterial repertoires should offer a comprehensive metric for clustering bacteria on the basis of their overall functional similarity–a combination of heritage and habitat impact.

We defined the functional repertoires of over 1,300 fully sequenced bacteria using protein clustering by HSSP (Homology-derived Secondary Structure of Proteins) distance [15]. HSSP techniques allow annotating two proteins as performing the same molecular function, without specifically defining the nature of this function. We also annotated our set of bacterial proteins via common function profiling tools: COG [16], Pfam [17], and RAST [18]. For the purposes of this work, we defined the similarity between any two organisms according to the percentage of functions they shared. We first validated the reliability of our functional similarity metric by using pairwise organism comparison to assign taxonomic ranks. Using the NCBI Taxonomy as a benchmark, we show that functional similarity, defined using any of the above-mentioned function annotation methods, is more descriptive of pairwise organism similarity than gene sequence identity–a novel finding. Additionally, our HSSP-based organism similarity metric was more accurate than metrics based on other function assignments evaluated in this study. Since HSSP is not limited by availability of annotations, our approach circumvents experimental limitations by including novel lesser-studied functions into organism classification.

We further identified natural clusters of bacteria in our functional-repertoire similarity-based organism network (FuSiON; flattened to *fusion*). Instead of assigning organisms into phylogeny-based classes, each of which may encompass a wide range of environmentally, metabolically, and phenotypically diverse microbes, *fusion* groups them according to functional similarity. Our scheme allows for variability in the number of non-hierarchical organism modules, where the clustering resolution is adjustable to each specific application. Moreover, as *fusion* is inherently cut-off free, its clade assignments are largely independent of current database biases, *i*.*e*. our method will not tend to assign a novel microbe to *Proteobacteria* simply because a vastly larger and more diverse set of *Proteobacteria* genomes are available in our databases. We investigated the functional basis for some of the individual discrepancies between the current taxonomy and the *fusion* classification via case studies in *Cyanobacteria* and *Mycoplasma*. We describe how phylogenetically related bacteria can still be functionally very different, with the environment playing a key role in selecting for each organism’s functional specificity. Our novel phenetic method for unambiguous and consistent classification of bacteria provides a complementary view to phylogenetic clade assignment. The dynamic nature of our network-based organism clustering provides an easy route for incorporation of additional organisms and organism features (*e*.*g*. plasmids) into the existing classification framework. *Fusion* is, thus, a more practical fit for biomedical, industrial, and ecological applications, *e*.*g*. [19,20], as many of these rely on understanding the functional capabilities of the microbes in their environment, and are less concerned with phylogenetic descent.

We are currently working on implementing a publicly available *fusion* work-bench, that will allow real-time assignment of novel organisms to *fusion* clades. All organism similarity and clustering data described in this work, along with the software and commands necessary to reproduce the reported *fusion* networks, are available for academic use and reuse under an open source license at: http://bromberglab.org/?q=services.

## Results and Discussion

### HSSP-based functional repertoire similarity accurately measures pairwise bacterial relationships

We annotated functions of 4.2 million proteins, encoded in 1,374 fully sequenced bacterial genomes, via COG, RAST, and Pfam. We also computed HSSP distances for every protein pair (~1.6x10^13^ comparisons). The HSSP distance is a non-linear metric incorporating sequence identity and alignment length that has been parametrized to identify alignments of proteins of experimentally established identical functions [15]. Briefly, enzymes of experimentally defined identical function (defined by the Enzyme Commission [21]) were used to determine a threshold curve separating the alignment length *vs*. sequence identity space into regions of same *vs*. different functions; *i*.*e*. two proteins that fall above the curve share identical function, while those below the curve do not. The distance of every alignment along the sequence identity axis away from the curve (HSSP distance) reflects the reliability of these assignments of functional identity [15].

We adopted an HSSP distance cut-off of 10, which annotates two proteins as sharing the same function with over 90% precision (accuracy/specificity, percentage of correct same-function predictions of all such predictions made), albeit at only ~40% recall (coverage/sensitivity, percentage of correct same-function predictions of all same-function pairs in the set) [15]. At this stringency, ~900,000 proteins (21% of 4.2 million in our set) were unique–one protein per functional group. The remaining 3.3 million clustered into ~335,000 functional groups (S1 Table). Note that at lower HSSP cut-offs these groups can be further consolidated, but at a significant loss of accuracy. We choose a more conservative threshold to attain maximal resolution of assignment.

We used RAST annotations to divide our HSSP-based functional groups into *Kn* (known; available annotation), *Hy* (hypothetical; likely protein existence, function not annotated) and *Un* (unknown; no annotation) sets (S1 Table; Methods). We further confirmed that each HSSP-based function group contained proteins of similar RAST annotations (S2 Table). Note that different function groups may contain proteins that carry out the same biochemical functions but in a different fashion, *e*.*g*. at different reaction rates. We found that many organisms contain proteins performing the *Kn* functions, while the *Hy* and *Un* functions tend to be organism specific, a conclusion that holds even if groups containing a single protein are excluded (S1 Fig). As a corollary, proteins carrying functions that are more common across organisms are more likely to be annotated (S1 Fig). Interestingly, we note that 26% (127,254 of 481,913) of the unannotated proteins in our set fall into the *Kn* (78%) and *Hy* (22%) HSSP-based function groups. We also show that for 71% of *Kn* groups (S2 Table), 90–100% of annotated proteins in each group are functionally identical. Our protein clustering may thus help elucidate functions of tens of thousands of yet un-annotated proteins; we anecdotally confirmed some of these via manual curation of new sequence annotations.

We defined the functional repertoire of an organism as the set of all functional groups carried by the organism. The size of the repertoire is at most as large as the number of proteins in the proteome, but in-paralogs may fall into a single functional group. The functional similarity of two bacteria was calculated as the number of shared function groups normalized by the bigger repertoire size (Methods).

Our HSSP-based functional group comparison significantly (Wilcoxon rank-sum test, *p-value*<0.0001; Methods) more accurately recapitulates the NCBI taxonomic identity of organism pairs than using other function definitions (COG, RAST, and Pfam) at all taxonomy levels, except the genus and species, where RAST achieves comparable performance (S2A–S2F Fig) RAST’s and HSSP’s improved performance at these lower levels may be due to their “whole sequence”-based function annotation. Pfam works at the domain level, which is arguably too broad, including many proteins into one function class. COG is designed to detect orthology, *i*.*e*. evolutionary relationships, and thus its functional groups are likely too narrowly defined. HSSP’s exemplary performance over all taxonomic levels is possibly due to the lack of dependence of its pairwise sequence comparisons on the external knowledge, *e*.*g*. Pfam domains, RAST functions, or COGs. Note that here we used COG instead of the more complete EggNOG [22], as we felt that manual curation may carry more resolution. We obtained the latest set of COG annotations from its developers (2012 update, Yury Wolf personal communication). Here we show that *all* tested function-based metrics reflect the current taxonomic organism placement fairly well. We adopt HSSP for this work as it correlates best with the current taxonomy (S2A–S2F Fig), while circumventing limitation of available protein function annotations.

As described above, the HSSP metric is more informative of function than protein sequence identity and alignment length alone [15]. Thus, although our method is mechanistically similar to sequence-based gene content phylogenomic approaches [23,24], it is very different from the latter both (1) conceptually–we classify organisms based on their current functional similarity rather than reconstructing their phylogeny and (2) practically–functional similarity significantly more accurately describes bacterial relationships than sequence identity-based methods (Wilcoxon rank-sum test *p-value*<0.0001; S2G Fig). The latter finding is intuitive, as function-based methods separate sequence-similar out-paralogs into different families, which sequence-based methods, by definition, cannot do. However, to the best of our knowledge the improvement of functional comparisons over gene content in classifying bacteria has not been experimentally shown before.

We find, perhaps unsurprisingly, that two nearly functionally identical (90% similarity) organisms belong to different species as often as a third of the time (S2F Fig). These functionally similar, yet taxonomically split organism pairs are not uniformly distributed throughout the taxonomy [10,25]. Here we show that most of these occur in three pathogenic genera: *Borrelia* (Lyme disease), *Brucella* (brucellosis), and *Mycobacterium* (leprosy, tuberculosis), suggesting possible bias of classification towards higher resolution for organisms of human interest. This preference is also evidenced by the relatively large number of experimental annotations of functions of the human-associated microbiome (S3 Fig) Though such taxonomic resolution bias probably offers convenience in practice, it brings along an inconsistency that complicates *en bulk* analysis of microorganisms; *e*.*g*. computational methods cannot readily deal with the type of subjectivity that separates very similar organisms into different taxa (*e*.*g*. *Borrelia hermsii* and *Borrelia turicatae* share 99% functional similarity), while assigning different organisms into the same taxon (*e*.*g*. *Clostridium botulinum* strains share less than 40% similarity). We argue that for practical use, it is often more important to know whether two organisms can perform the same molecular functions rather than if they share the same lineage.

Note that throughout this work, in order to compare our organism assignments to the current taxonomy, we conservatively excluded the plasmid proteomes. Plasmids contribute heavily to functional differentiation, as opposed to speciation, separating classes of microorganisms without explicit phylogenomic commitment. Moreover, plasmids follow independent evolutionary models [26,27] and carry many of the environment-related functions [28]. We expect that including the plasmid genomes into our paradigm will show stronger impact of habitat and we intend to evaluate plasmid contribution in further work.

### *Fusion* organism classification correlates with the NCBI Taxonomy

**Fig 1:**
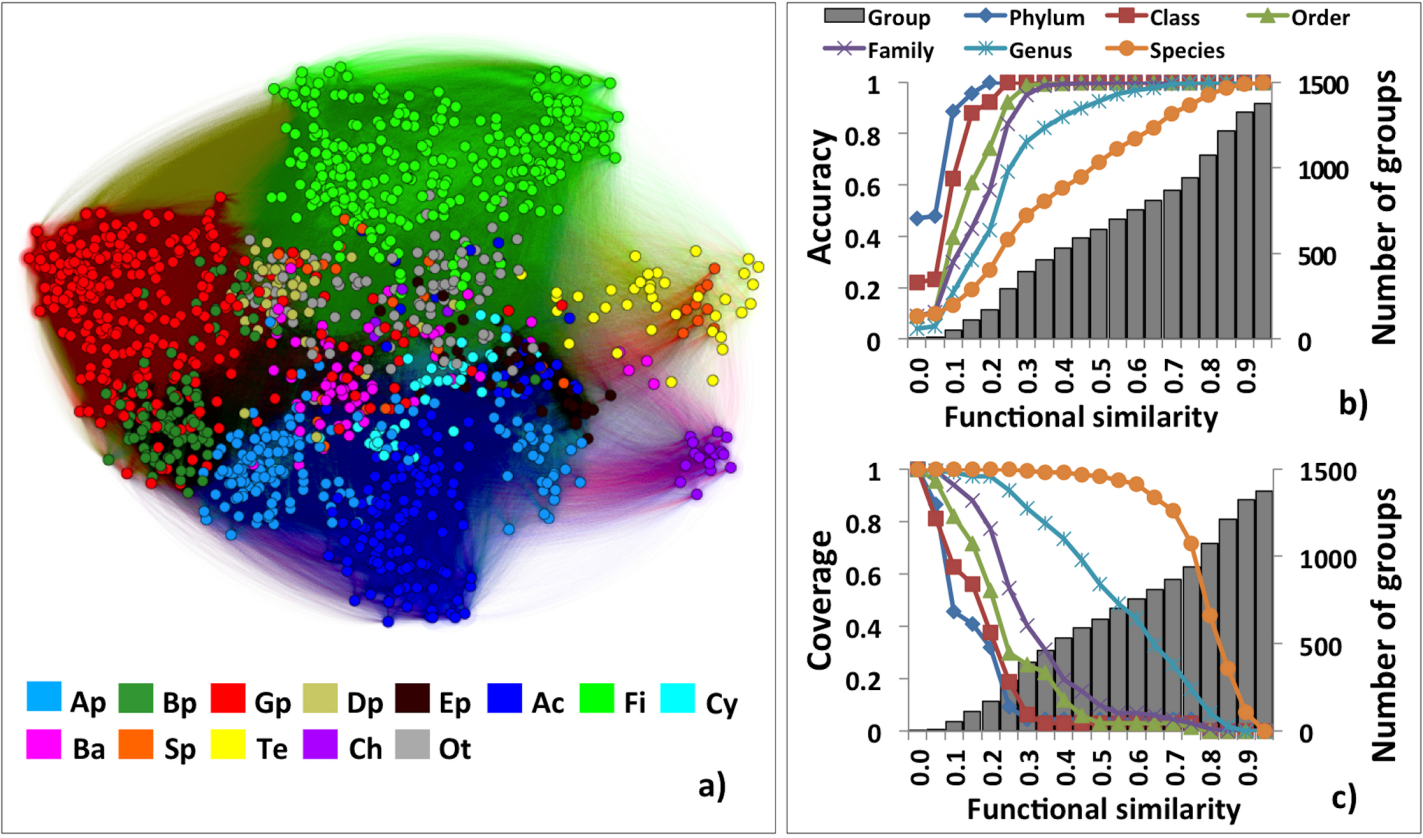
*Fusion*-based clustering correlates with NCBI Taxonomy. (**a**) *fusion* network colored by taxonomic rank. Ap-*Alphaproteobacteria*; Bp-*Betaproteobacteria*; Gp-*Gammaproteobacteria*; Dp-*Deltaproteobacteria*; Ep-*Epsilonproteobacteria*; Ac-*Actinobacteria*; Fi-*Firmicutes*; Cy-*Cyanobacteria*; Ba-*Bacteroidetes*; Sp-*Spirochaetes*; Te-*Tenericutes*; Ch-*Chlamydiae*; Ot-other minor phyla; (**b**) The overall accuracy of functional similarity networks at cut-offs from 5% to 100%, with step of 5%. The overall network accuracy is the fraction of correctly assigned organisms of the total number of organisms; *i*.*e*. overall accuracy of 100% indicates that all organisms in any one cluster are of the same taxon. The overall accuracy for each taxonomy level increases with the cut-off. Thus, lower taxonomy levels (e.g. genus, species) achieve 100% overall accuracy at higher cut-offs; (**c**) The overall coverage of the functional similarity networks at cut-offs from 5% to 100%, with step of 5%. The overall coverage is the percentage of taxa (excluding taxonomic singletons) with all members in one cluster at a given cut-off. Overall coverage of 100%, indicates no splitting of any of the taxa; *i*.*e*. one cluster per taxon. Lower taxonomy levels lose 100% overall coverage at higher cut-offs.

We represented the functional similarity of our microorganisms as a network–*fusion* (functional-repertoire similarity-based organism network). In *fusion*, organisms are vertices (nodes), and edge lengths (weights) indicate pairwise functional repertoire similarities. Here all organisms (1,374 nodes) are at least somehow similar forming a fully connected network (943,251 edges). The minimum amount of similarity between two organisms is <1%—these edges link the tiny *Candidatus* microbes (S3 Table) to the much bigger organisms in our set. However, the most common level of similarity between two organisms is 7% (mean 7.7% and median 6%). These results indicate that our organisms are mostly functionally distant, but maintain a minimal set of identical, globally present, likely housekeeping, functions. In a representation that takes into account edge-weight and node density (Fig 1A; OpenORD layout [29]), microorganisms cluster consistently within their NCBI Taxonomy groups.

Earlier studies searched for natural discontinuity in the bacterial pairwise genome similarity space [30,31], but found no unique break point that would reasonably assign taxa to large sets of organisms. To inspect for possible occurrence of these breakpoints in our network representation, we adopted a range of cut-offs in a single linkage clustering approach (S1 Text). With increasing cut-offs, our network contained organism clusters that were progressively more taxonomically consistent at lower taxonomic ranks (S4A–S4C Fig). This split into clusters is informed by the variation in density of organisms across the network, *i*.*e*. the increased connectivity between nodes within one region as compared to outside the region. Note that density is artificially increased in regions of preferentially studied organisms (e.g. *Firmicutes* and *Proteobacteria*, S3 Fig). To study the mapping of functional relationships to taxonomy, we used 1% cut-off increments in the network to build a 100-layer hierarchical structure (Methods; S4D Fig). We found that this structure was somewhat topologically similar (corr = 0.557) to the NCBI Taxonomy. However, the differences between the two (S1 Data) indicated the absence of natural breakpoints correlating the current taxonomy to functional groupings of microorganisms.

To quantify the cluster-taxon consistency, we calculated the overall network accuracy and coverage at different cut-offs (Methods). With the cut-off increasing from 5% to 100%, the overall accuracy increases while the overall coverage decreases for each taxonomy level (Fig 1B and 1C). Note that the 100% overall accuracy for the species level is only attained at 100% cut-off, which results in one organism per cluster (Fig 1B); *i*.*e*. NCBI Taxonomy assigns highly similar organisms into different species. On the other hand, even 10% functional similarity does not guarantee 100% overall coverage for most (phylum to genus) taxonomic levels (Fig 1C). All strains of a single species consistently fall into a single cluster (100% overall coverage) only until the 30% cut-off; *i*.*e*. highly dissimilar organisms are classified into the same species.

The lowest cut-off resulting in 100% overall accuracy, along with the highest cut-off resulting in 100% overall coverage, define lower and upper bounds, respectively, of the functional repertoire similarity in assigning NCBI Taxonomy. Organisms in different clusters at cut-offs less than the lower bound are of different taxa, while organisms in the same cluster at cut-offs greater than the upper bound are of the same taxon. The ranges of uncertainty of taxonomic assignment (region between the lower and upper bound) are varied and often large, *e*.*g*. spanning cut-offs of 5–95% for genus-level classification (S5A Fig). Pairwise comparisons (S5B Fig) display similar behavior, highlighting inconsistencies in the prokaryotic taxonomy, previously quantified by *e.g. [25]*. Arguably, even more disconcerting for pairwise comparisons is the fact that >90% of all organism pairs fall into this uncertainty range for all taxonomic ranks except for species (most organism pairs are of different species); *e*.*g*. for phylum level 97% of all organism pairs are in the uncertainty region. These results indicate that setting arbitrary cut-offs, whether network- or pairwise- comparison-based, in order to fit organisms into preset taxonomic bins, inevitably introduces unquantifiable and non-standardizable bias into annotations–a problem for large-scale organism and microbiome studies.

### *Fusion* modules reflect non-hierarchical organism groupings

State of the art in any field often concerns itself with describing available data points and extrapolation on the basis of observed trends. Current prokaryotic taxonomy is, thus, primarily defined on the basis of culturable and commonly studied microorganisms, *e*.*g*. *Proteobacteria* and *Firmicutes*, which make up 46.8% and 21.7% of our data set, respectively. Furthermore, the number of well studied organisms of a particular kind is often the driving force of taxonomic placement of newly discovered (sequenced) organisms; *i*.*e*. you could only compare a new organism to existing ones, so better represented clades are more likely to be populated with additional members. For example, when looking to classify a newly cultured microbe on the basis of 16S rRNA gene sequence similarity, one is simply more likely to find a closer, even if not sufficiently close, sequence belonging to a well studied clade than to a poorly described one. Re-assignment of organisms to new clades on the basis of additional evidence is fairly common. However, follow-up studies are time consuming, limited to organisms of high interest, and, thus, unlikely to find all errors. High-throughput experimental methods (*e*.*g*. cheaper sequencing) and automated organism classification can contribute to further propagation of assignment errors. An unfortunate, but highly visible result of this state of the art is the significant difference in annotations of organism diversity of the same metagenomic sample using data provided by different 16S rRNA databases [32].

Network-based organism similarity representations can help alleviate issues of data availability bias. In a fully connected network of similarities, non-overlapping modules, with denser (edge weight-wise) within-module and sparser across-module connectivity, imply natural organism grouping. The Louvain algorithm [33] maps nodes in a network into modules by considering both edge-weight (extent of similarity) and node connectivity. When all-to-all connectivity exists within a network, edge-weight is the sole driver of module detection; *i*.*e*. five very similar organisms can form a module of their own as well as ten or twenty organisms. In fact, a larger number of organisms is more likely to connect strongly outside the module and, thus, be subject to dispersion. A newly identified organism, placed into a fully connected network is then subject to forces (connections) pulling from all directions, to finally identify its placement. This placement is dynamic–as new organisms are added a network’s partitioning can change. As a result, this approach is more robust to dealing with natural organism diversity than static structures.

For our purposes, one big advantage of the Louvain algorithm is that it splits the fully connected *fusion* network into communities (modules) without a need for a set arbitrary similarity cut-off. However, a problem with this single best grouping of organisms is that when the global modularity function is optimized, there is a loss of resolution for smaller modules. An adapted version of the Louvain method [34], instead of modularity, aims to optimize stability of network partitions over time. Here, stability reflects flows of probability through the network, capturing important aspects of the global architecture and describing different optimal partitions of the network at different times. Simply put, a module is considered stable if random walkers (described by a particular Markov process [34]) do not escape from it within the set time limit. Thus, longer time limits (higher “resolution” parameter values (S6 Fig) result in larger and coarser (more functionally diverse) modules. The size and diversity of organism modules can thus be optimized for each individual application.

While one may see the resolution parameter as cut-off equivalent, it is in fact quite different. In setting cut-offs on organism similarity we consistently group organisms within the same hierarchy–two organisms of the same species always belong to the same genus and the same phylum. On the other hand, tuning the stability of modules is a dynamic assignment. Thus, two organisms in a low-resolution module can belong to different modules at medium resolution and the same module again at high resolution. Note that this implementation of Louvain algorithm is not deterministic; that is two organisms (at the “edge” of similarity) can be sorted into different modules with two runs of the algorithm at the same resolution setting. Correspondence of partitions (estimated by *e*.*g*. [35]) produced at the same resolution setting can thus be used to approximate meaningful partition points for growing *fusion* networks as new organisms are added. This option is not available for similarity cut-off-based schemes that are easily skewed by the availability of genomic data, which, for now, is heavily biased toward organisms of particular human interest (S3B Fig). Though *fusion* is also affected by genome availability, the effect is alleviated by all-to-all connectivity, which reduces the importance of node number in favor of edge weight for clustering purposes.

**Fig 2:**
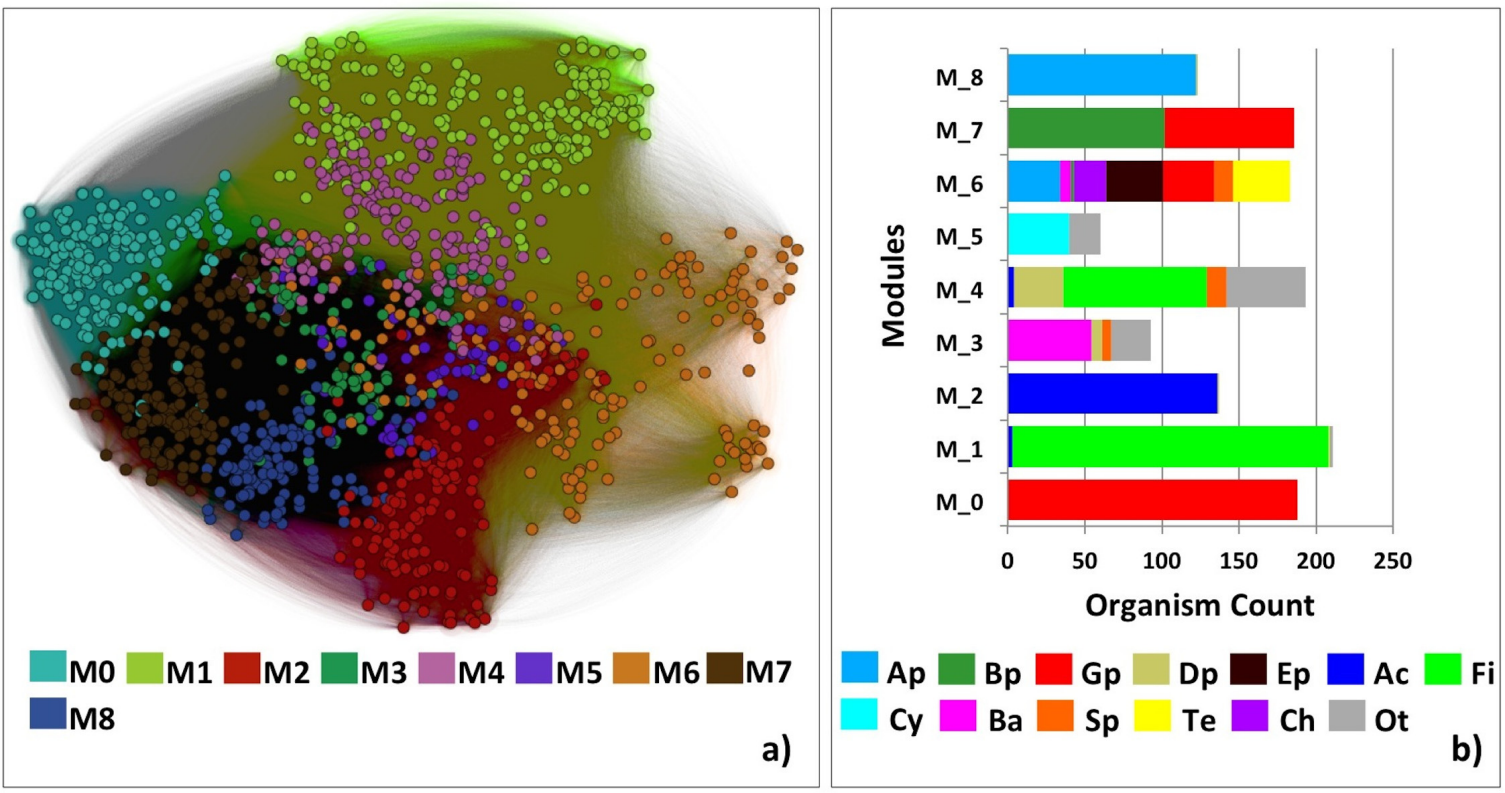
*Fusion* module detection reveals natural organism grouping. **(a)** Colors represent each of the nine *fusion* modules detected at resolution 0.8. **(b)** Organism diversity (NCBI Taxonomy) in each module is shown as: Ap-*Alphaproteobacteria*; Bp-*Betaproteobacteria*; Gp-*Gammaproteobacteria*; Dp-*Deltaproteobacteria*; Ep-*Epsilonproteobacteria*; Ac-*Actinobacteria*; Fi-*Firmicutes*; Cy-*Cyanobacteria*; Ba-*Bacteroidetes*; Sp-*Spirochaetes*; Te-*Tenericutes*; Ch-*Chlamydiae*; Ot-other minor phyla. The difference in diversity among the different modules reflects the inconsistencies of the current taxonomy.

We detected the Louvain communities in the complete *fusion* network (no edges removed) using a set of resolution values. We compared organism pair assignments to the same Louvain community *vs*. the same NCBI taxonomic placement using the Jaccard index (species to phylum; resolution 0.05 to 1.2; Table 1 and S6 Fig). Here this metric (ranged [0,1], from “no similarity” to “identity”) evaluates the percentage of organism pairs that is simultaneously assigned to the same module and the same taxonomic clade, of all same module or same clade assignments (Methods). For example, at the 0.8 resolution of *fusion* (Fig 2A; colors indicate modules) there are nine modules detected. The NCBI taxon (class for *Proteobacteria* and phylum for all others) of organisms in these modules varies (Fig 2B). Some modules demonstrate a highly homogeneous phylum/class distribution, while others are diverse. The Jaccard index of this resolution is 0.478 with NCBI class assignment and 0.294 for phylum assignment. This observation highlights the inconsistency of functional microorganism abilities with the current taxonomic assignments.

**Table 1:** Similarity of the NCBI Taxonomy assignments and *fusion* modules.

	Modularity index	Number of *fusion* Modules	Number of NCBI clades	Jaccard index
**Phylum**	1.1	3	27	0.423
**Class**	0.8	9	43	0.416
**Order**	0.5	56	97	0.611
**Family**	0.4	99	204	0.433
**Genus**	0.3	170	493	0.458
**Species**	0.1	551	875	0.177

We suggest that our novel network-based classification scheme reveals the natural grouping of organisms instead relying on arbitrary similarity cut-offs. Unlike classification based on *pairwise* organism similarity, *fusion* is more robust in handling microorganism diversity. It also alleviates the data availability (organism bias) problem and is a more practical fit for large-scale computational analysis. In addition, without the limitation of preset discrete taxonomic bins, users can zoom in/out with different resolutions to find out the functional organism groups of their specific interest.

### *Fusion*+ reveals functional basis of classification discrepancy

To study the functional basis of taxonomic vs. functional discrepancies, we built, for several cases, a variant of the *fusion* network, *fusion*+. Our case studies were *Mycoplasma* and *Cyanobacteria*–organisms with well-known taxonomy assignment issues (Garrity GM 2001). *Fusion*+ has two types of nodes: organisms and functions that they perform. Organism nodes are connected by edges to their function nodes. Thus, while in *fusion* one edge connects each organism pair, in *fusion*+ the number of connecting edges is equal to the number of shared functions. Thus, *fusion* modules can be studied in depth in terms of specific functions or organism meta-data variables, *e*.*g*. salinity, temperature, or pH preferences.

### *Mycoplasma* studies

**Fig 3:**
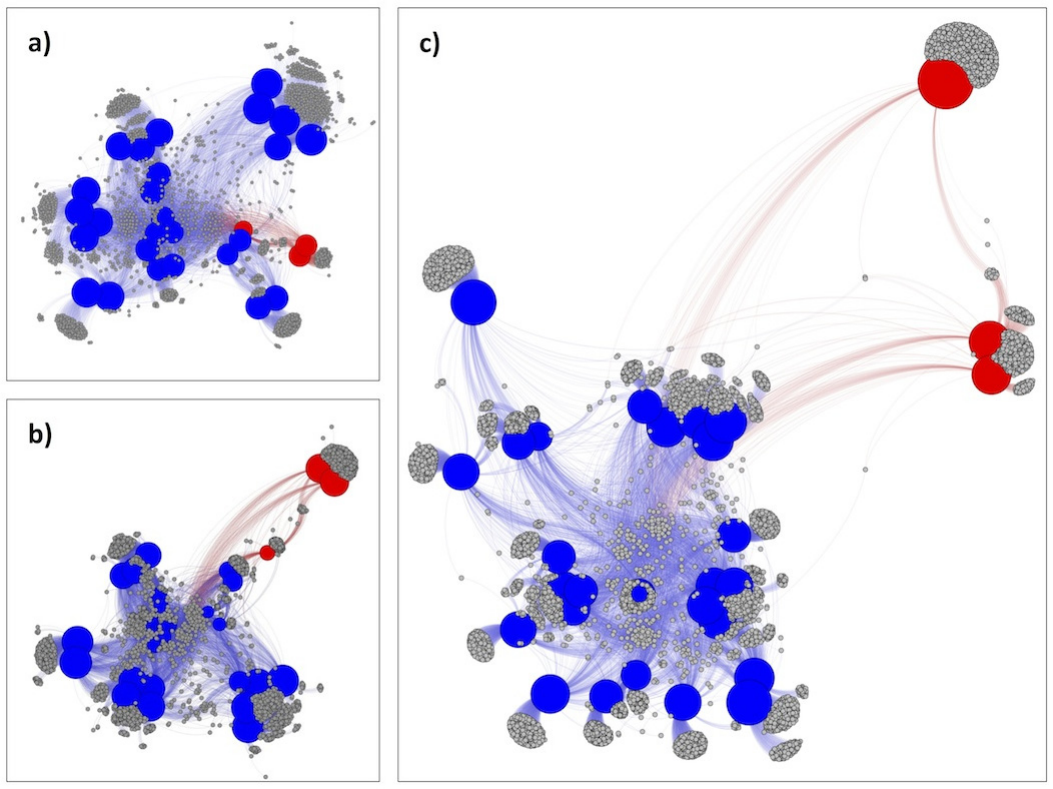
*Mycoplasma fusion*+ reveals the importance of *Hy* and *Un* functions in taxonomy assignment. The networks include a) *Kn* functions, b) *Kn* and *Hy* functions and c) all functions. Unique blood *Mycoplasma* organisms are indicated by red nodes, with the rest of *Mycoplasma* colored in blue. The length of edges represents the relative (not absolute) similarities between organisms. Note the resolution increases as *Hy* and *Un* functions added.

We created three *fusion*+ networks for 29 *Mycoplasma* strains, including (1) only their 1,848 *Kn* functions (Fig 3A), (2) 1,848 *Kn* and 1,347 *Hy* functions (3,195 total, Fig 3B), and (3) all 9,354 functions (Fig 3C). The shift of the *M*. *suis* and *M*. *haemofelis* Langford 1 away from other *Mycoplasma* between *Kn*-only (Fig 3A) and *Kn*,*Hy*-network (Fig 3B) illustrates the importance for classification of the yet unstudied (*Hy*) functions. Note that while adding the 1,518 *Un* (95% organism-unique) functions further increases the separation between all organisms in the network (Fig 3C), this effect can be largely attributed to the impact of repertoire size.

The separation of the two *M*. *suis* strains and *M*. *haemofelis* from other *Mycoplasma* is not surprising. As noted earlier, in the functional similarity network they form isolated clusters at a very low 10% cut-off (S4A Fig and S3 Table). Previously known as *Eperythrozoon suis* and *Haemobartonella felis*, respectively, these three strains were moved to the *Mycoplasma* genus on the basis of their 16S rRNA phylogeny [36,37]. There is, however, ample biological differences of these strains as compared to other *Mycoplasma* [38]. Quantifying these differences is, however, very difficult–do they merit re-assignment to another clade or not? Our observations highlight the problem: these organism are assigned into a genus with less than 10% of common functionality–even organisms of different phyla are often more similar (S4A Fig). The structure of the *fusion* network, however, clearly groups them with other *Mycoplasma* all the way down to a resolution of 0.1. While the similarity of *fusion* modules and species assignments is fairly low (Table 1), in this particular case the two metrics agree. Rooted in the same ancestor as other *Mycoplasma*, *M*. *suis* and *M*. *haemofelis* have evolved specific functional differences likely due to their unique epierythrocytic parasitic life styles [39]. However, in the currently available microbial functional landscape, even these (very dramatic) in-clade differences do not make this set of organisms functionally different enough to merit complete clade dispersal. This example demonstrates the subjective (albeit successful, in this case) nature of current cladistic assignments when evolutionary relatedness does not equal functional similarity.

We further identified 26 (25 *Kn* and one *Hy*) functional groups shared between *M*. *suis* and *M*. *haemofelis*, but not by other *Mycoplasma* (S4 Table). Representative sequences from two of these groups are detected in a variety of other organisms from multiple phyla. The rest are exclusive to *M*. *suis* and *M*. *haemofelis*. Note that other organisms carry out the biochemical functions represented by these functional groups, but they do so using sufficiently different proteins from the ones specific to these *Mycoplasma* strains. These differences may include different protein stabilities, different rates of reaction, *etc*. For instance, many of these 25 *Kn* function groups are house-keeping; *e*.*g*. DNA polymerase subunits that are unlike others in our set, indicate a likely ancient split from other *Mycoplasma*.

One difference between *M suis* and *M*. *haemofelis* is their preferred hosts, swine and feline, respectively. The species differ from each other by 1,686 functions – 640 in *M*. *suis* (88% unique; remaining 79 functions shared with other *Mycoplasma*) and 1,046 in *M*. *haemofelis* (98% unique). This finding is in line with the fact that many hemotrophic *Mycoplasma* contain numerous paralogous gene families, which are thought to participate in antigenetic variation [40]. These functions are less annotated, but likely differentiate these organisms in ways necessary to evade specific host immune response.

### *Cyanobacteria* studies

**Fig 4:**
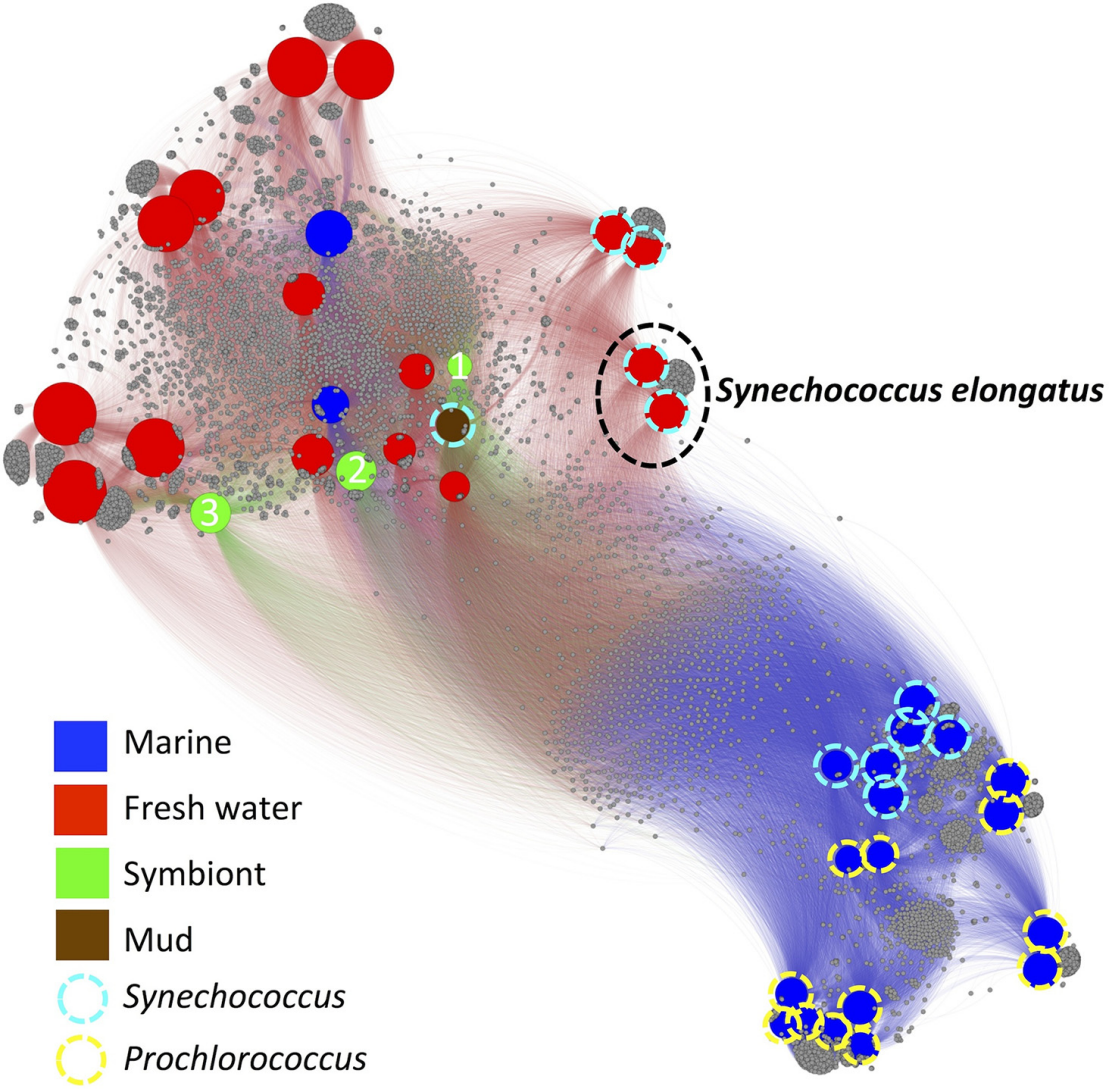
*Fusion*+ of 40 *Cyanobacteria* reveals environment impact on functions. The *Cyanobacteria* form one mostly fresh water cluster and one marine cluster. The members of *Synechococcus* exist in both clusters. The functions that are shared between marine *Synechococcus* and *Prochlorococcus*, yet not found in fresh water *Cyanobacteria*, are likely important in the marine environment. Symbiont1-cyanobacterium UCYN-A; Symbiont2- *Acaryochloris marina* MBIC11017; Symbiont3- *Nostoc azollae* 0708.

We explored the *fusion*+ network of 40 *Cyanobacteria* (49,937 functions: 17,275 *Kn*, 21,465 *Hy*, 11,197 *Un*; 34,678 organism unique). Based on the 15,259 functions shared by at least two organisms, the *Cyanobacteria* separate into two clusters (Fig 4). In *fusion* this split is observed at resolution 0.3 –a genus equivalent. One cluster (Fig 4, top) contains 16 fresh-water *Cyanobacteria*, three symbionts [41–43], two marine-water organisms and one isolated from marine mud. Note that the mud dweller, *Synechococcus* PCC 7002, is salt tolerant, but does not require salt for growth [44]. Another cluster (Fig 4, bottom) contains only marine *Cyanobacteria*. The *Synechococcus* genus members are found in both clusters with marine *Synechococcus* sharing more functionality with the marine *Prochlorococcus* than with the fresh water *Synechococcus*. The intra-genus diversity of *Synechococcus* [44] suggests a division into five genera-equivalent subgroups [10]. *Fusion*+ reveals that the fresh water and marine *Synechococcus* are significantly functionally different and should belong to different taxa, an unsurprising finding that is in line with both 16S rRNA-based phylogenetic [45] and phylogenomic [46] studies. Bergey’s Manual relies heavily on morphology for *Cyanobacteria* classification. However, for this specific example using phylogeny would produce more informative taxonomic assignments. In other cases, phylogeny may be misleading. For example, according to evolutionary ancestry fresh-water *Synechococcus elongatus* strains should group together with the marine *Synechococcus* and *Prochlorococcus* [45,46]. However, *S*. *elongatus* is more functionally similar to fresh water *Synechococcus* (Fig 4) and should be grouped with them despite its evolutionary relationships to the marine subgroup.

To further study salt tolerance, we identified 181 functional groups only shared by the marine *Synechococcus* and *Prochlorococcus* in our network. Of these, 15 groups include proteins from organisms of various phyla; *e*.*g*. one of these functions is present in *Allochromatium vinosum*, a halotolerant microbe surviving in both marine and freshwater environments [47]. This particular function is RAST annotated as a putative carboxysome peptide A, crucial in carbon fixation. We hypothesize that this *A*. *vinosum* version of the carboxysome subunit is either specific to salt adaptation or transferred together with other salt tolerance genes in an HGT event. We also identified 166 functions (including 21 *Hy* and one *Un* function) exclusive to and ubiquitous in the marine *Synechococcus* and *Prochlorococcus* (S2 Data). Of these, 34 were unique–not found in any other organisms (including the closest evolutionary neighbor, *S*. *elongatus)* in any other form (manual curation).

### Functional similarity can standardize organism classification

*Fusion* offers a quantitative, objective, and consistent function-based measure of organism similarity. Its classifications correlate with the current taxonomy for many organisms, but not in cases where close phylogenetic relatives are functionally different. Our analysis supports previously reported trends of inconsistencies in the current taxonomy [30,31]. *Fusion*’s functional repertoire definitions are more accurate for organism classification than sequence identity-based whole-genome comparisons. Moreover, our novel *network* scheme with module identification, to the best of our knowledge, is the first attempt to highlight naturally occurring clusters of organisms without (arbitrary) pairwise similarity cut-offs. It is more robust than pairwise organism comparison in dealing with organism diversity, particularly since much of *fusion*’s resolution comes from using unstudied (or poorly studied) functions. Potentially, its use of functional similarities to identify organisms can facilitate organismal and functional diversity annotation of metagenomes and, under some circumstances, even contamination detection in newly sequenced genomes. *Fusion* reveals the significant roles that environmental factors play in determining functional abilities of organisms and highlights the key functions shared by different organisms in the same environment.

For large-scale analyses and practical applications requiring systematic organismal phenotype assessments, *e*.*g*. antibiotics development, bioremediation, and industrial uses, classification based on functional comparisons may carry more meaning than evolutionary relationships. *Fusion* is a novel framework for organism classification that (1) directly uses organism functional comparisons, eliminating the need to consider individual HGT events in addition to evolutionary lineage, (2) describes organismal diversity by identifying natural organism clusters in a similarity network instead of arbitrarily establishing cut-offs in levels of similarity per cluster, and (3) has an unlimited capacity to incorporate additional genetic data from plasmids and/or previously unseen organisms. At the very least, *fusion* offers a complementary view to the current taxonomy. Comparing the two classification schemes allows detection of functionally diversified strains–an ability that, potentially, has a wide range of applications, *e*.*g*. tracking and surveillance of bacterial pathogens.

### Conclusion

Microorganism classification, like many other scientific strategies, is driven by expertise and available technology. Historically designed with more emphasis on the former, the current taxonomy lacks consistency across assignments. Recent advances in sequencing abilities have created the possibility of exploiting entire organism functional pools for classification. Here we demonstrate *fusion*–a classification technique that compares molecular (genome encoded) functionality across microorganisms. *Fusion* can be used with a predictable consistency to classify newly sequenced organisms according to the current taxonomy. More importantly, it offers a novel and practical prokaryotic classification scheme, which is reflective of, but not dependent on, organism evolutionary history. *Fusion*’s ability to highlight functions key to particular environments will have great impact in industrial and clinical practices.

## Methods

### Datasets

We downloaded 1,374 bacterial proteomes from December 2011 NCBI GenBank release [48]. Habitat information for these organisms was obtained from GOLD [49] and IMG [50].

### Defining functional repertoires and their similarity

We defined the functional repertoire of a single microorganism to be the set of all molecular function capabilities carried by its proteome (excluding plasmids).

#### HSSP-based protein clustering

We performed an all-to-all PSI-BLAST [51] of 4.2 million protein sequences in the 1,374 bacteria proteomes (parameters: e-value 1e^-3^; inclusion ethresh 1e^-10^; num iterations 3; max target seqs 1e^9^; num alignments 1e^9^). HSSP distances [15] were calculated from the PSI-BLAST results (Eq 1), where *L* is the length of the alignment between two proteins and *Id* is the percentage of identical residues.

HSSPdistance={-99,L<11Id-480L-0.32(1+e-L1000),11<L≤450Id-19.5,L>450 (1)

The highest HSSP distance was selected for every pair of proteins when multiple alignments were possible. Note that here higher distance means higher similarity. A threshold of HSSP distance ≥10 was used to define two proteins as having similar function. At this threshold, the HSSP metric attains ~90% precision and ~40% recall in mapping functional identity of protein pairs [15]. We further clustered these proteins into function groups using MCL (Markov Cluster Algorithm; parameter:-I 1.4) [52].

#### Other function profiling tools

We obtained COG (Clusters of Orthologous Groups) [16] annotations for our dataset (personal communication with Dr. Yuri Wolf). We downloaded the Pfam database (release 27.0) [17] and annotated all proteins using hmmscan [53] against both PfamA and PfamB with default settings. We kept the top hit for each protein with e-value < 1e^-3^. We used a local install of the RAST toolkit (myRAST) [18] to annotate the function of all proteins. Each annotation was made at the default reliability level (parameters:-reliability 3). All the proteins that were not annotated by COG, Pfam and RAST were counted as representing individual functions.

The *functional repertoire similarity* of two organisms was calculated as the number of shared functions in each functional repertoire (as defined by different tools above) divided by the bigger repertoire size. We assumed that similar organisms should have similar repertoire sizes, thus a vast difference indicates low similarity.

For comparison to gene content phylogenomic approaches, we also calculated the *whole-genome similarity* as the number of shared homologous proteins (homology inferred via 40% sequence identity) normalised by the bigger proteome size.

### Annotation of function groups derived from HSSP-based protein clustering

We divided all 4.2 million proteins in our set into three categories based on their RAST annotation: 1) *known*, sequences with available function annotation; 2) *hypothetical*, sequences with “hypothetical” or “putative” in their annotation, or annotated as “protein” or “Uncharacterized protein conserved in bacteria,” and 3) *unknown*, sequences with no annotations at all. We further assigned all of our HSSP-based function groups to one of three categories; for a given function group: 1) *Kn* if it contains at least one sequence of the *known* category; 2) *Hy* if it contains no *known* sequences and at least one *hypothetical* sequence and 3) *Un* if it contains only unknown sequences. In addition, we also tagged our function groups as 1) *shared*, if they exist in more than one organism in the dataset or 2) *unique*, if they exist only in one organism.

### Comparing the performance of the different pairwise similarity metrics to infer organism taxonomy

For every pair of organisms of known NCBI Taxonomy identity [48], functional repertoire similarities were computed using annotations from COG, Pfam, RAST, and our HSSP-based method. Each method provided either (i) a correct assignment to the same taxon (true positive, TP), (ii) an incorrect assignment to the same taxon (false positive, FP), (iii) a correct assignment to different taxa (true negative, TN), or (iv) an incorrect assignment to different taxa (false negative, FN). The accuracy (positive accuracy, precision; PA) and coverage (positive coverage, recall; PC) were computed for every metric at every threshold (Eq 2). We then compared the taxonomic classification performance of the different functional repertoire similarity metrics and the whole-genome similarity.

$$PA=\frac{TP}{TP+FP}\;PC=\frac{TP}{TP+FN}$$ (2)

Bootstrap analysis was performed by randomly sampling 10% of the data with replacement 100 times for each taxonomy level. AUC (Area Under the Curve) under the accuracy/coverage (precision/recall) curve was calculated [54] for every functional similarity metric and Wilcoxon rank-sum tests were performed for every pair of metrics.

### Generating functional-repertoire similarity-based organism networks

*Fusion* and *fusion*+ networks were visualized using Gephi [55] OpenORD [29] and ForceAtlas2 [56], respectively.

In *fusion* each 1,374 organisms (vertices/nodes) are connected by 943,251 edges whose weights reflect the pairwise organism functional repertoire similarities. In *fusion*+ vertices/nodes represent organisms and function groups. A (larger) organism node shares edges with its (smaller) function group nodes. Organism nodes are linked to each other only via function group nodes; *i*.*e*. there is no edge directly linking organism nodes. The common function group nodes are between organism nodes, while the unique function nodes tend to localize near the edges of the network.

### Calculating overall accuracy and coverage for singly linked networks

In single linkage clustering any two nodes that share an edge are assigned to a single cluster regardless of their similarity to other nodes in that cluster. The presence of an edge indicates similarity of organisms above a minimum cut-off, but the level of similarity is not further considered. Isolated organisms, with no connection to any other organism in our set, were not shown.

We measured the performance of single linkage clustering in identifying current taxonomic assignments for a series of similarity cut-offs (5%-100%, at step of 5%, Fig 1B and 1C). For each cut-off, we assigned all organisms in one single linkage cluster to the taxon of the most common organisms in that cluster; *e*.*g*. if a cluster of three organisms contained two organisms of taxon X, all three were assigned to the taxon X. The overall network accuracy was calculated as the sum of all the correctly assigned organisms divided by the total number of organisms (Eq 3).

$$OverallAcc=\frac{\sum_{i=1}^{n}correctly\;assigned\;organisms\;in\;cluster\;i}{total\;number\;of\;organisms}$$ (3)

We also identified the organism clusters consistent with taxonomic assignments of their members; *e*.*g*. if 7 organisms are assigned to a taxon X, and 4 of them are in cluster A, then A is considered the *major* cluster of X. For each taxon, the coverage is the fraction of its members that are in the *major* cluster (Eq 4); *e*.*g*. for X in our example coverage is 57%. At 100% coverage all members of a taxon are in one cluster. For a given taxonomy level, the overall network coverage was calculated as the number of taxa with 100% coverage divided by the total number of taxa at this level (Eq 5). Note that taxa with only one member would contribute trivially to the performance, and thus were excluded for these calculations.

$$Cov=\frac{Organisms\;in\;the\;major\;cluster}{Total\;number\;of\;organisms\;in\;the\;taxon}$$ (4)

$$OverallCov=\frac{Taxa\;with\;100\%\;coverage}{Total\;number\;of\;taxa\;with\;more\;than\;one\;organism}$$ (5)

### Comparing single linkage functional network-based organism classification to the NCBI Taxonomy

The 100-layer network-derived hierarchy was built by starting at the threshold of 0% functional repertoire similarity, *i*.*e*. all 1,374 bacteria are in a single cluster, and moving outward in 1% increments until the 100% similarity threshold was reached. For a given cluster of organisms sharing at least X% similarity, we (i) clustered the organisms at (X+1)% similarity, (ii) calculated the distance between every two clusters by computing the average of all inter-cluster pairwise similarities of organisms and (iii) built a neighbor-joining tree (layer) of the clusters using PHYLIP [57]. By combining all layers we obtained a 100-layer hierarchical tree-like structure. This hierarchical structure provides a compact visual representation of functional similarity of our large groups of microorganisms. Note, however, that it is not a phylogeny tree and does NOT directly convey organismal evolutionary relationships.

NCBI Taxonomy hierarchical tree-like structure was generated with iTOL [58] using the NCBI Taxonomy IDs [48]. We then computed the correlation (ranged -1 to 1) between network and NCBI-derived hierarchical structure using Patristic [59]. The hierarchical structures were first converted to distance matrices in which the distance between two organisms was calculated as the steps between them in the hierarchy. We also built 6 and 10 layer network-derived structures to show that the difference in the number of layers is not relevant to the comparison of the topological *relative* distances of any two organisms across hierarchies.

### Detection of *fusion* modules and calculation of Jaccard index

We identified modules in the complete (no similarity cut-offs) *fusion* with the adapted Louvain method [34] implemented in Gephi at a series of resolutions (0.05 to 1.2). We further calculated the Jaccard index to compare organism assignments from *fusion* modules to the NCBI Taxonomy. At a given resolution, the Jaccard index is calculated as the number of organism pairs assigned to both the same *fusion* module and the same NCBI Taxonomy bin, divided by the number of organism pairs assigned to either the same *fusion* module or the same NCBI Taxonomy bin.

## Supporting Information

S1 Text Mapping cut-off based single linkage clusters to current taxonomy. (DOCX)

S1 Fig Function groups that are shared by many organisms are more likely to be experimentally annotated (Kn>Hy>Un). (TIFF)

S2 Fig HSSP-based functional similarity correlates with the NCBI Taxonomy better than other function definitions. (TIFF)

S3 Fig Bias in functional annotation of bacterial proteomes. (TIFF)

S4 Fig Functional network single linkage clustering correlates with NCBI Taxonomy. (TIFF)

S5 Fig Both network-based single linkage clustering and pairwise functional repertoire similarity correlate poorly with NCBI Taxonomy. (TIFF)

S6 Fig Organism pairs assigned to the same *fusion* module seldom overlap with pairs assigned to the same NCBI Taxonomy bin. (TIFF)

S1 Table Annotation status of HSSP-based function groups. (DOCX)

S2 Table Distribution of proteins of the same functional annotation among all the HSSP-based function groups. (DOCX)

S3 Table Six bacteria not matching any organisms in the functional repertoire-based network at 10% cut-off. (DOCX)

S4 Table Blood Mycoplasma functional groups different from other Mycoplasma. (DOCX)

S1 Data Major discrepancies between *fusion* single linkage clusters and the NCBI taxonomy. (XLSX)

S2 Data Marine *Synechococcus/Prochlorococcus* functions different from other *Cyanobacteria*. (XLSX)
