# Supplementary material for: Functional Basis of Microorganism Classification
Source: PLoS Comput Biol. 2015 Aug 28;11(8):e1004472. doi: 10.1371/journal.pcbi.1004472 (PMC4552647; doi:10.1371/journal.pcbi.1004472)
Supplement: S1 Text — (DOCX) [file pcbi.1004472.s001.docx]

Supporting Text

S1 Text

**Mapping cut-off based single linkage clusters to current taxonomy.** In single linkage clustering any two nodes that share an edge are assigned to a single cluster regardless of their similarity to other nodes in that cluster. The presence of an edge indicates similarity of organisms above a minimum cut-off, but the level of similarity (edge weight) is not further considered. Thus, a large and diverse set of organisms could form a single cluster at fairly high similarity cut-offs; *i.e.* if the similarity cut-off is 15% and organism A is 20% similar to organism B, while B is 20% similar to organism C, then all three organisms are assigned to the same cluster even if A and C share less than 10% similarity. At the 10% cut-off, *i.e.* when a minimum of 10% repertoire similarity creates an edge between two organisms, four clusters were formed, encompassing 1,368 (99% of all) organisms (S4A Fig.). Note that at this 10% cut-off, we removed the majority of the edges in our network (~86%). As expected from a large and diverse group, 1,355 organisms fell into one cluster. The other three clusters contained a total of 13 organisms, including five of the *Planctomycetes* phylum in one cluster, six of the *Leptospira* genus in another, and two *Mycoplasma suis* species strains in the third. The separation of *Planctomycetes* can be explained by the uniqueness of this phylum ([Fuerst and Sagulenko 2011](#_ENREF_1)). However, the split of *Leptospira* away from other genera of *Spirochaetes*, as well as the split of *Mycoplasma suis* and *Mycoplasma haemofelis* Langford 1 from each other and other *Mycoplasma,* highlight the (known) disagreements of the current taxonomic clade assignments with these organisms’ functional abilities ([Garrity GM 2001](#_ENREF_2)). Note, however, that *Spirochaetes* and *Tenericutes* (to which *Mycoplasma* belong) make up less than 2% of our set, each. Thus, their functional split could also suggest experimentally determined lack of similar genomes. The six singletons, *i.e.* organisms sharing less than 10% functional similarity with any other organisms in our dataset, are summarized in S3 Table. Individuality of some of these can be explained – *Fibrobacter succinogenes* S85 is the only *Fibrobacteres* member in our dataset, as may be the three *Candidatus* organisms of unusually small repertoire sizes. However, the reasons for differentiating *Bdellovibrio bacteriovorus* HD100 from its taxonomic neighbors must be rooted in the dissimilarity of functional annotations and taxonomic assignments.

References

Fuerst JA, Sagulenko E (2011). Beyond the bacterium: planctomycetes challenge our concepts of microbial structure and function. *Nature reviews Microbiology* **9:** 403-413.

Garrity GM BD, Castenholz RW, editors (2001). *Bergey's Manual of Systematic Bacteriology, Volume 1*, 2nd edn. Springer: New York (NY).
