# Supplementary figures and images for: Functional Basis of Microorganism Classification
Source: PLoS Comput Biol. 2015 Aug 28;11(8):e1004472. doi: 10.1371/journal.pcbi.1004472 (PMC4552647; doi:10.1371/journal.pcbi.1004472)

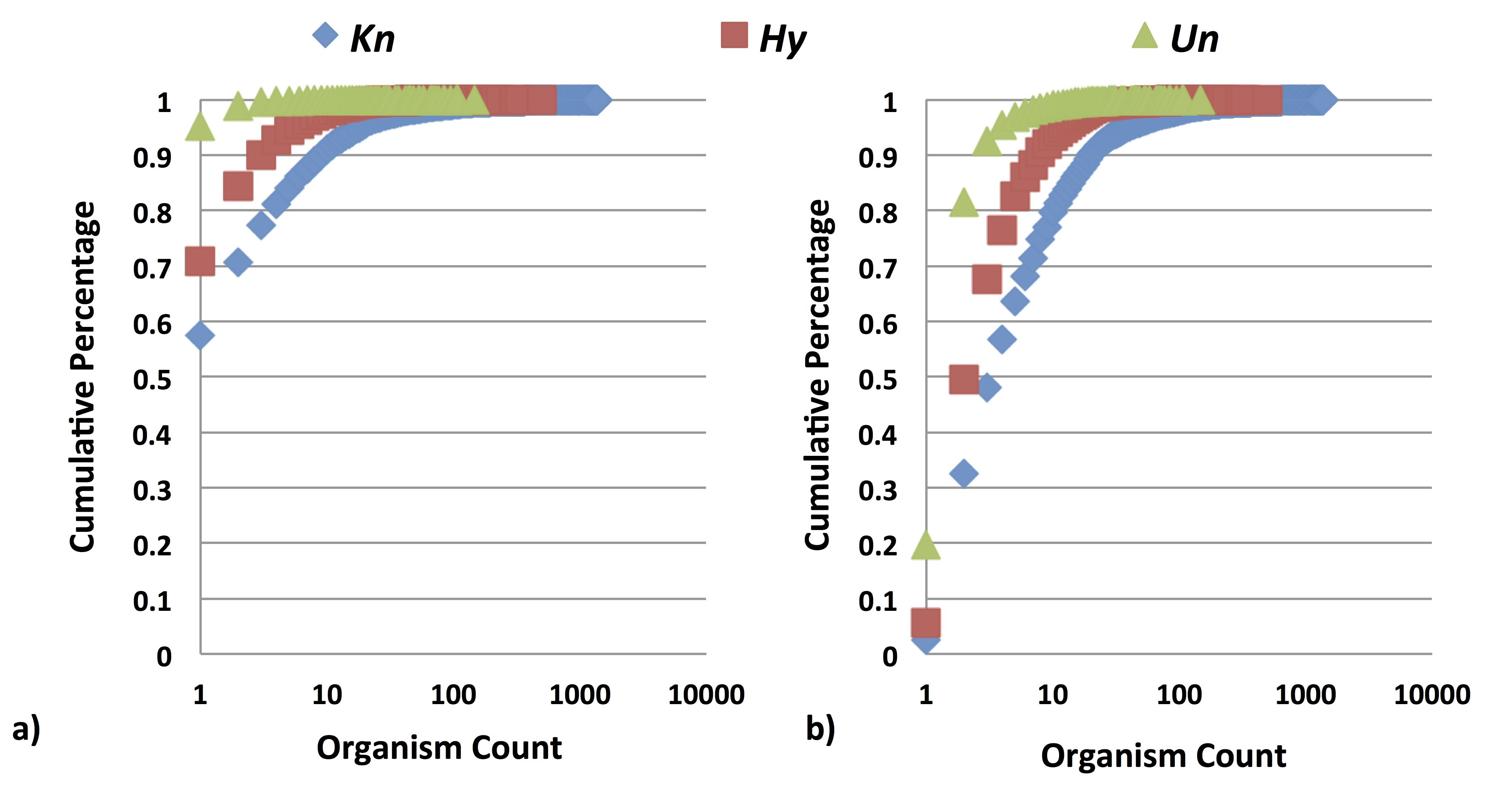

Supplement: S1 Fig — (TIFF) [file pcbi.1004472.s002.tiff]

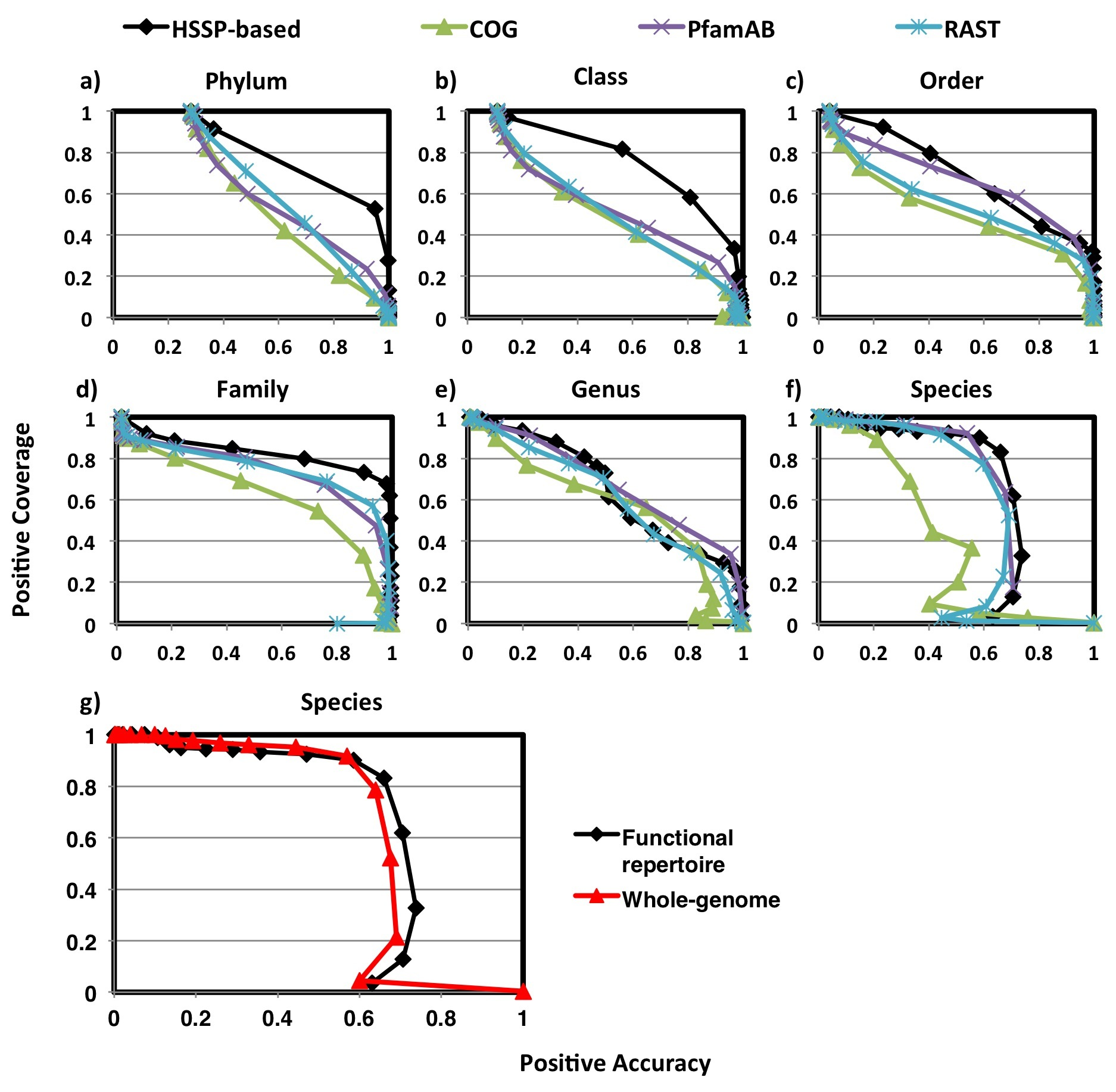

Supplement: S2 Fig — (TIFF) [file pcbi.1004472.s003.tiff]

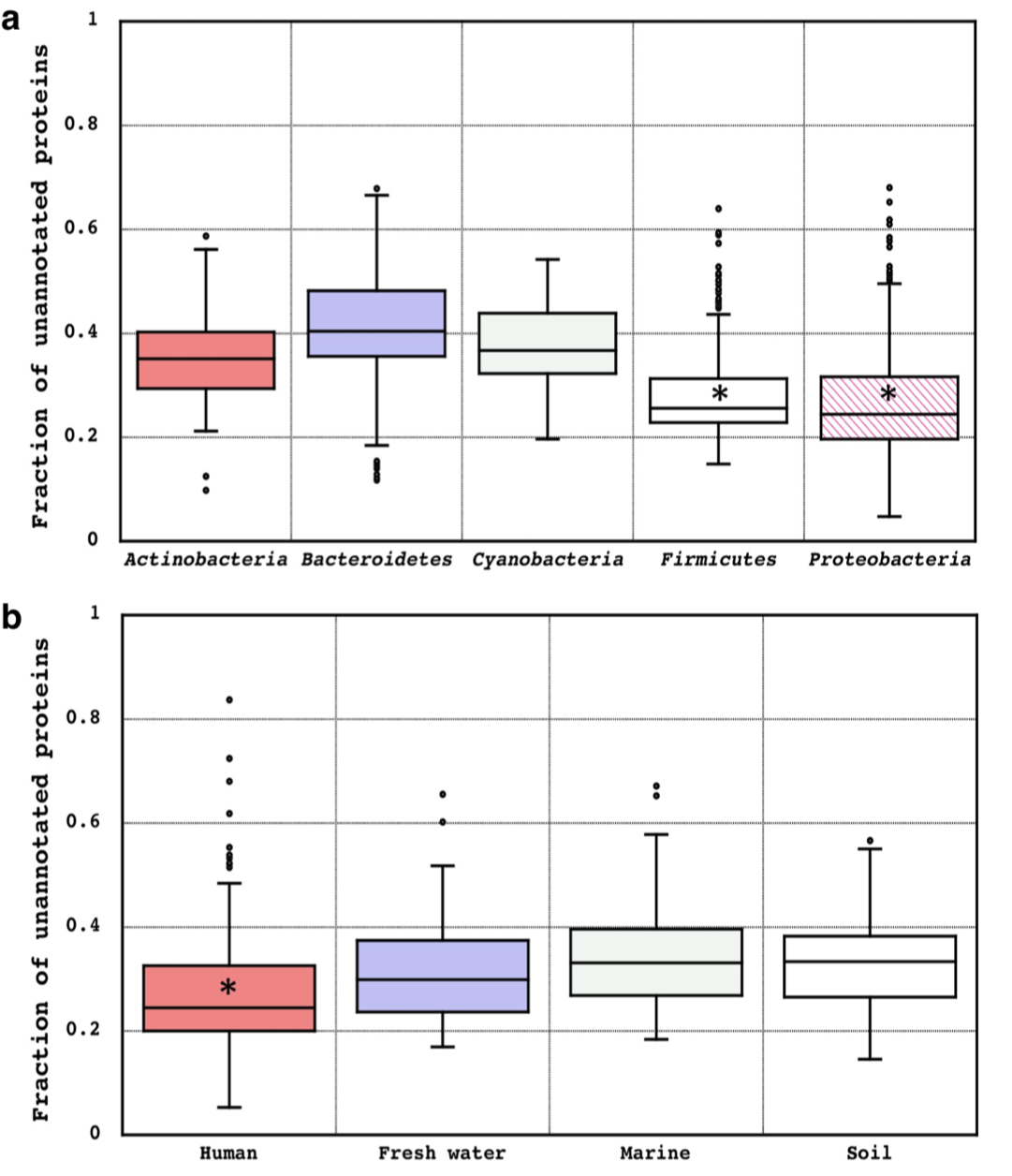

Supplement: S3 Fig — (TIFF) [file pcbi.1004472.s004.tiff]

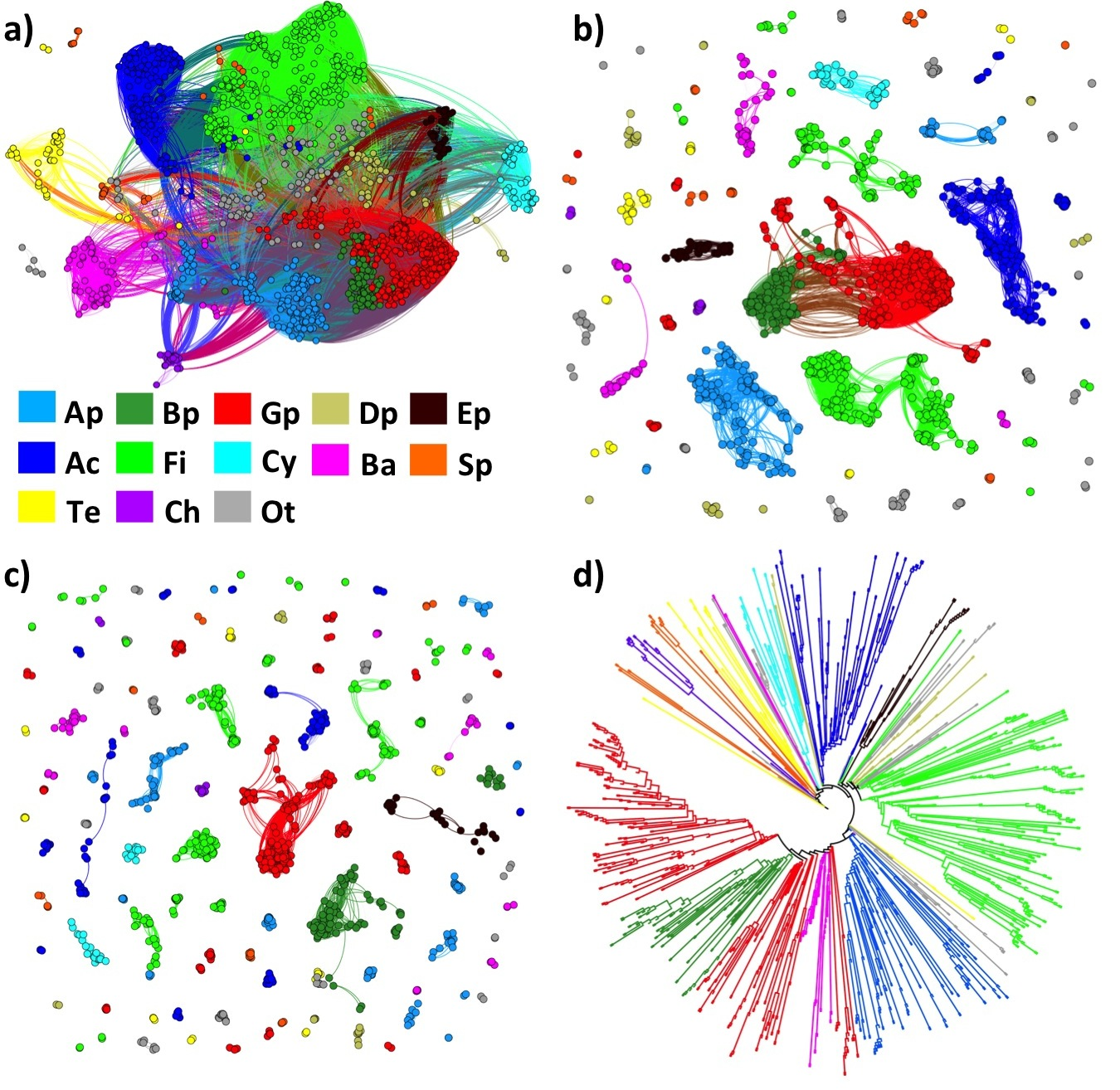

Supplement: S4 Fig — (TIFF) [file pcbi.1004472.s005.tiff]

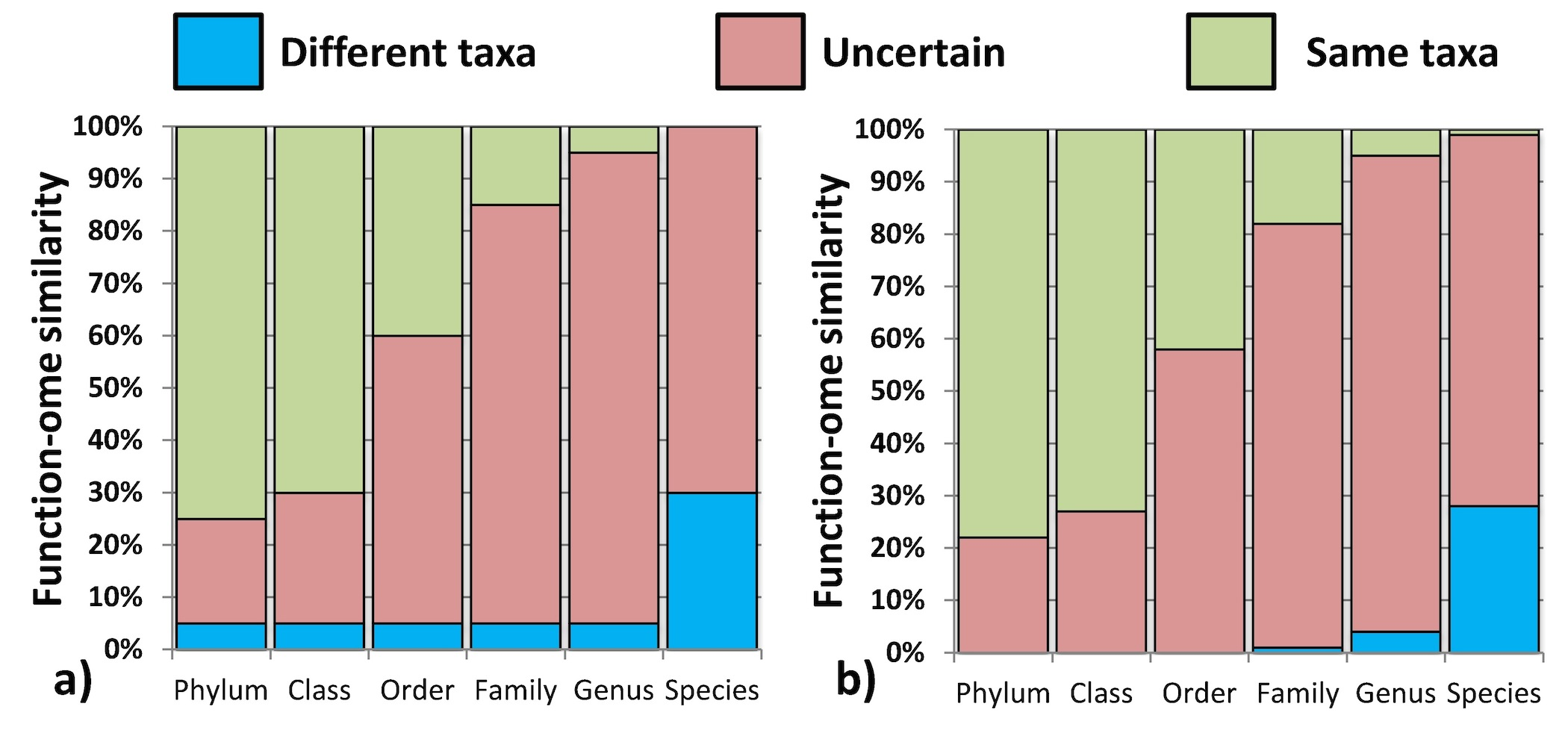

Supplement: S5 Fig — (TIFF) [file pcbi.1004472.s006.tiff]

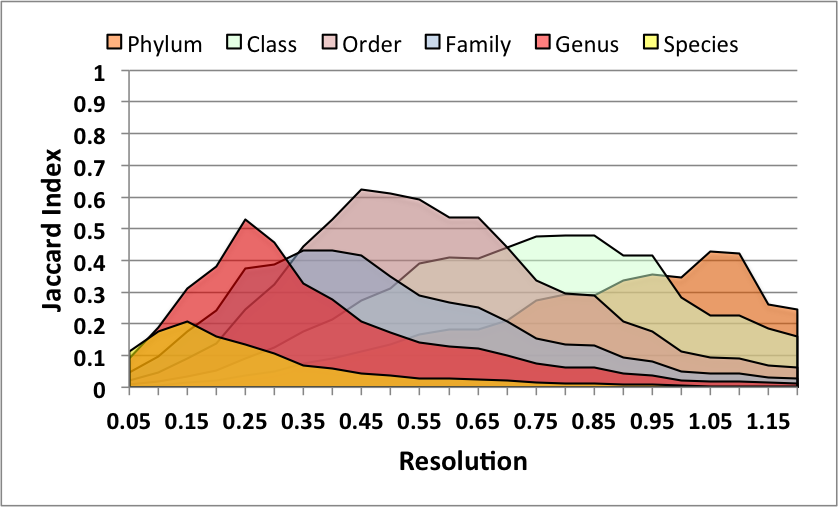

Supplement: S6 Fig — (TIFF) [file pcbi.1004472.s007.tiff]
