# Supplementary material for: Functional Basis of Microorganism Classification
Source: PLoS Comput Biol. 2015 Aug 28;11(8):e1004472. doi: 10.1371/journal.pcbi.1004472 (PMC4552647; doi:10.1371/journal.pcbi.1004472)
Supplement: S1 Table — (DOCX) [file pcbi.1004472.s008.docx]

S1 Table. Annotation status of HSSP-based function groups.

|  | Function groups (>1 sequence) | Function groups (1 sequence) | Total |
| --- | --- | --- | --- |
| Known (Kn) | 190,272 | 245,430 | 435,702 |
| Hypothetical (Hy) | 119,825 | 267,160 | 386,985 |
| Unknown (Un) | 24,925 | 387,768 | 412,693 |
| Total | 335,022 | 900,358 | 1,235,380 |
