# Supplementary material for: Functional Basis of Microorganism Classification
Source: PLoS Comput Biol. 2015 Aug 28;11(8):e1004472. doi: 10.1371/journal.pcbi.1004472 (PMC4552647; doi:10.1371/journal.pcbi.1004472)
Supplement: S2 Table — (DOCX) [file pcbi.1004472.s009.docx]

S2 Table. Distribution of proteins of the same functional annotation among all the HSSP-based function groups.

| Proteins with same annotation  (% of all in a group) | # of groups (% of total) |
| --- | --- |
| 0-50 | 12,777 (6.7) |
| 50-90 | 41,634 (21.9) |
| 90-100 | 135,861 (71.4) |
