# Supplementary material for: Functional Basis of Microorganism Classification
Source: PLoS Comput Biol. 2015 Aug 28;11(8):e1004472. doi: 10.1371/journal.pcbi.1004472 (PMC4552647; doi:10.1371/journal.pcbi.1004472)
Supplement: S3 Table — (DOCX) [file pcbi.1004472.s010.docx]

S3 Table. Six bacteria not matching any organisms in the functional repertoire-based network at 10% cutoff.

| Organism | Functional Repertoire Size  (# of groups) | Phylum/Class |
| --- | --- | --- |
| Bdellovibrio bacteriovorus HD100 (uid61595) | 3,426 | Deltaproteobacteria |
| Candidatus Carsonella ruddii (uid58773) | 181 | Gammaproteobacteria |
| Candidatus Hodgkinia cicadicola Dsem (uid59311) | 168 | Alphaproteobacteria |
| Candidatus Tremblaya princeps PCIT (uid68741) | 119 | Betaproteobacteria |
| Fibrobacter succinogenes S85 (uid41169) | 2,837 | Fibrobacteres |
| Mycoplasma haemofelis Langford 1 (uid62461) | 1126 | Tenericutes |
