# Supplementary material for: Functional Basis of Microorganism Classification
Source: PLoS Comput Biol. 2015 Aug 28;11(8):e1004472. doi: 10.1371/journal.pcbi.1004472 (PMC4552647; doi:10.1371/journal.pcbi.1004472)
Supplement: S4 Table — (DOCX) [file pcbi.1004472.s011.docx]

S4 Table. Blood *Mycoplasma* functional groups different from other *Mycoplasma*.

| Cluster | #Seq | #Bacteria | Annotation |
| --- | --- | --- | --- |
| C_1 | 128 | 122 | DNA-cytosine methyltransferase (EC 2.1.1.37) |
| C_2 | 82 | 77 | Ribonucleotide reductase of class Ib (aerobic), beta subunit (EC 1.17.4.1) |
| C_3 | 4 | 3 | DNA polymerase III subunits gamma and tau (EC 2.7.7.7) |
| C_4 | 3 | 3 | DNA polymerase III alpha subunit (EC 2.7.7.7) |
| C_5 | 3 | 3 | Glucose-6-phosphate isomerase (EC 5.3.1.9) |
| C_6 | 3 | 3 | Cardiolipin synthetase (EC 2.7.8.-) |
| C_7 | 3 | 3 | RecA protein |
| C_8 | 3 | 3 | SSU ribosomal protein S7p (S5e) |
| C_9 | 3 | 3 | Inosine-5'-monophosphate dehydrogenase (EC 1.1.1.205) |
| C_10 | 3 | 3 | Thioredoxin reductase (EC 1.8.1.9) |
| C_11 | 3 | 3 | FIG006542: Phosphoesterase |
| C_12 | 3 | 3 | tRNA (5-methylaminomethyl-2-thiouridylate)-methyltransferase (EC 2.1.1.61) |
| C_13 | 3 | 3 | Phospholipid-lipopolysaccharide ABC transporter |
| C_14 | 3 | 3 | Endonuclease IV (EC 3.1.21.2) |
| C_15 | 3 | 3 | LSU ribosomal protein L13p (L13Ae) |
| C_16 | 3 | 3 | Ferrichrome transport system permease protein FhuG |
| C_17 | 3 | 3 | Transcription termination protein NusA |
| C_18 | 3 | 3 | Preprotein translocase secY subunit (TC 3.A.5.1.1) |
| C_19 | 3 | 3 | SSU ribosomal protein S5p (S2e) |
| C_20 | 3 | 3 | SSU ribosomal protein S19p (S15e) |
| C_21 | 3 | 3 | 6-phosphofructokinase (EC 2.7.1.11) |
| C_22 | 3 | 3 | Tryptophanyl-tRNA synthetase (EC 6.1.1.2) |
| C_23 | 3 | 3 | Zn-dependent hydrolase (EC 3.-.-.-) |
| C_24 | 3 | 3 | hypothetical protein |
| C_25 | 3 | 3 | Adenylosuccinate lyase (EC 4.3.2.2) |
| C_26 | 3 | 3 | ABC TRANSPORTER PERMEASE PROTEIN |
